# An unusual intraoperative finding during tension-free vaginal tape surgery for stress urinary incontinence: a case report

**DOI:** 10.1093/jscr/rjag115

**Published:** 2026-03-07

**Authors:** Myriam Lili Ceballos Posada, Katherine Arias

**Affiliations:** Department of Urology, Fundación Valle del Lili, Cra 98 No. 18–49, Cali 760032, Valle del Cauca, Colombia; Universidad Icesi, Facultad de Ciencias de la Salud, Calle 18 No. 122–135, Cali 760031, Valle del Cauca, Colombia; Clinical Research Center, Fundación Valle del Lili, Cra 98 No. 18–49, Cali 760032, Valle del Cauca, Colombia

**Keywords:** stress incontinence, urethral sling, herniorrhaphy, surgical mesh

## Abstract

Stress urinary incontinence is commonly managed with tension-free vaginal tape (TVT), a highly effective mid-urethral sling procedure. We report the case of a 64-year-old woman with prior left inguinal herniorrhaphy who presented with urodynamically confirmed stress incontinence. During retropubic TVT placement, marked resistance was encountered on the left side, requiring multiple attempts before successful passage. Her postoperative course was uneventful. This unusual technical difficulty was likely related to fibrosis or mechanical interference from a prior groin mesh. The case underscores the importance of detailed surgical history and, when indicated, preoperative imaging to anticipate anatomical variations that may complicate sling placement.

## Introduction

Urinary incontinence (UI) is a common condition, particularly prevalent among older adults, and is associated with factors such as aging, obesity, multiparity, and prior urogynecologic surgery [1]. A cross-sectional study aimed at characterizing UI in a cohort of 13 383 individuals reported a prevalence of 8.8% (*n* = 1172), with a higher burden in women (11.2%) compared with men (5.5%) [2].

The most common subtypes of UI include stress urinary incontinence (SUI), urgency urinary incontinence, and mixed urinary incontinence. This report centers on SUI, whose prevalence among women older than 30 years ranges from 24% to 45% [3]. SUI is defined as the involuntary leakage of urine precipitated by increases in intra-abdominal pressure, such as during coughing, laughing, or sneezing and is primarily attributable to urethral hypermobility or intrinsic sphincter deficiency [4].

Initial management includes conservative measures such as weight reduction, modification of risk factors, pelvic floor muscle training, biofeedback therapy, and pharmacologic interventions [5]. Surgical treatment is considered in refractory cases. Available surgical options include Burch colposuspension, transobturator tape (TOT), and retropubic mid-urethral slings such as the tension-free vaginal tape (TVT), the latter being widely adopted due to their minimally invasive nature, low complication rates, and rapid postoperative recovery [5].

We present this case due to the absence of prior reports describing incidental intraoperative findings associated with inguinal hernia repair mesh.

## Case report

The patient was a 64-year-old woman with a history of urolithiasis and recurrent cystitis; prior left inguinal herniorrhaphy, cholecystectomy, hysterectomy at age 41, and endoscopic ureterolithotomy. Obstetric history was G2P1A1. She presented with progressive SUI classified as Blaivas type II [6], requiring daily pad use and occasionally diapers. Symptom onset occurred after her hysterectomy.

She reported increased daytime and nighttime urinary frequency, urgency incontinence, obstructive voiding symptoms, and suprapubic pain. Physical examination revealed pale vaginal mucosa without evidence of prolapse; bimanual examination showed no masses or tenderness, and pelvic floor muscle contraction was deficient. Urodynamic testing confirmed SUI (VLPP >100 cm H₂O).

**Figure 1:**
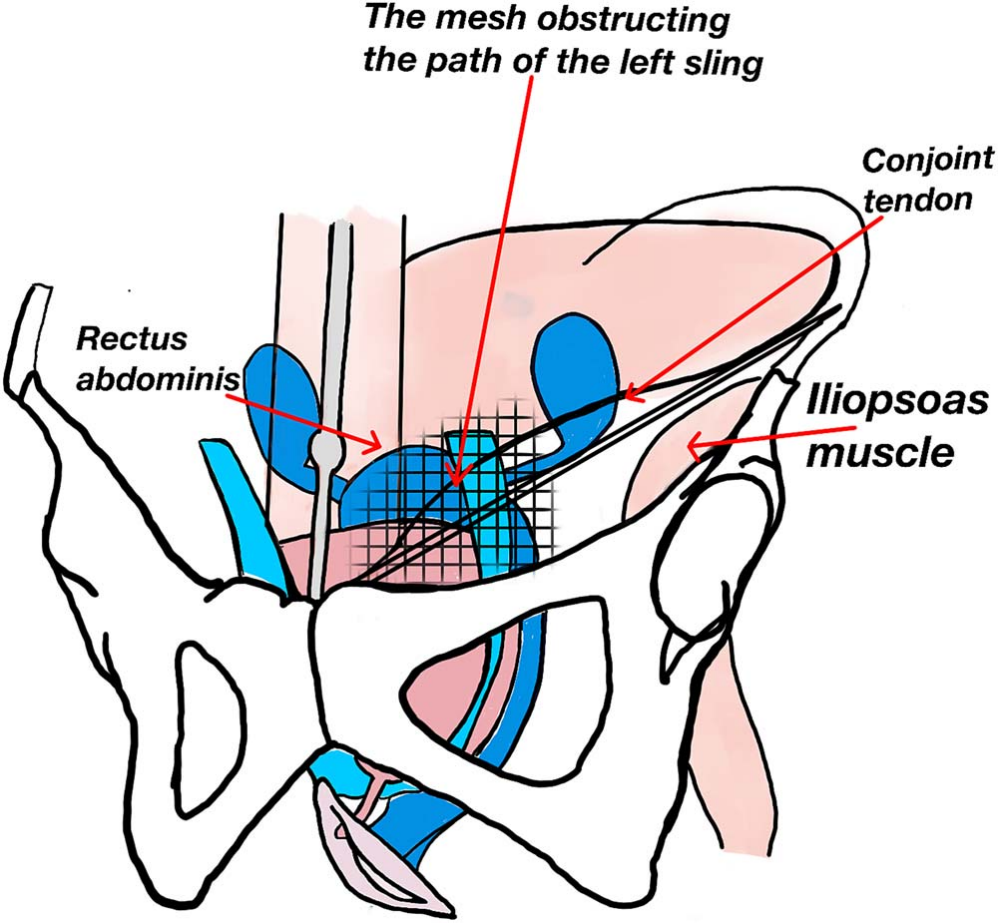
Illustration of the mechanical resistance encountered during left retropubic passage of the TVT sling.

**Figure 2:**
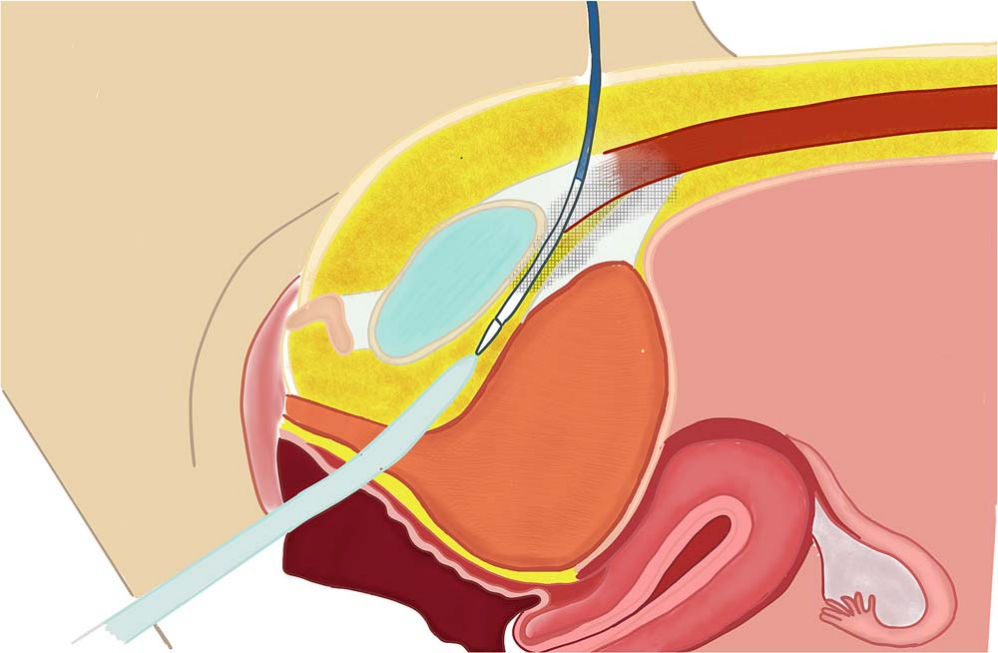
Sagittal illustration of sling passage interference.

Surgical management with retropubic mid-urethral sling placement was performed under general anesthesia. Advancement of the right-sided trocar proceeded without incident. In contrast, during the left-sided passage, substantial resistance was encountered (Fig. 1), causing repeated trocar deflection. After multiple attempts and assisted guidance using a precut silk suture, successful advancement into the retropubic space was ultimately achieved (Fig. 2). The postoperative course was uneventful, with only mild discomfort localized to the left iliac fossa.

## Discussion

Mid-urethral sling placement is the most commonly performed surgical treatment for SUI, with a well-established efficacy and safety profile. Although complications such as bladder perforation, voiding dysfunction, and vascular injury have been described, mechanical resistance to needle passage is exceedingly uncommon [7]. Schütze *et al*. [7] reported, in a retrospective study of 873 patients (TVT: 306; TOT: 567), an intraoperative complication rate of 3%, with bladder perforation being more frequent in the TVT group (2.6% vs. 0.2%). No instances of mechanical resistance were described.

The TVT technique involves the placement of a polypropylene sling beneath the mid-urethra through the retropubic space. A suburethral vaginal incision is first made, after which the sling is advanced toward the retropubic space on the right side; the same maneuver is then performed on the left, exiting at the suprapubic level. Both arms of the tape are left tension-free, allowing for fixation through fibrosis and integration with the surrounding tissues [8].

Approximately 10% to 15% of the global population either has or will develop an abdominal wall hernia. Among the various hernia types, inguinal hernias are the most common, accounting for nearly 60% of all abdominal hernias diagnosed. They predominantly affect men, with a male-to-female ratio of approximately 4:1 [9]. Surgical repair involves reduction of the hernia sac and, in the case of a direct hernia, reinforcement of the posterior inguinal wall. This can be accomplished using either the Bassini technique, where the transversus abdominis edge is approximated to the inguinal ligament or a mesh-based repair, in which a prosthetic mesh is placed over the transversalis fascia to cover the myopectineal orifice of Fruchaud [9].

Interference between the two procedures may occur because the TVT needle traverses the space of Retzius, passing near the internal inguinal ring, which corresponds to the site where reinforcement of the posterior inguinal wall is performed during hernia repair. If a large mesh or one with posterior extension was used, it may occupy the retropubic space (the same trajectory followed by the sling). Likewise, if the mesh was anchored to structures such as the Cooper ligament, the pubic symphysis, or the transversus abdominis muscle, it could generate mechanical interference during TVT needle passage [9]. Furthermore, mesh placement may induce fibrosis within the retropubic space, similarly hindering advancement of the tape. No imaging studies were available to determine the precise location of the mesh from the prior procedure. The likelihood of a woman having a history of inguinal herniorrhaphy is low [9], and consequently, the probability that such a repair could alter the normal course of TVT placement is even lower.

Only one case from Germany has been reported in the literature, in which technical difficulty occurred during retropubic sling insertion due to resistance on the right side. During postoperative recovery, the patient developed signs of acute blood loss, including tachycardia and hypotension, and was found to have an 800-cc clotted hematoma in the space of Retzius, located medial–lateral to the obturator nerve [10]. This case represents an intraoperative finding similar to ours, although it occurred in a patient without a history of prior inguinal hernia repair mesh.

Among the limitations, there are no comparable cases in the literature describing a similar technical difficulty to contrast with the findings of the present report. In addition, information regarding the surgical details of the prior inguinal herniorrhaphy is limited, as the procedure was performed outside our institution.

## Conclusion

This report describes an unusual technical difficulty encountered during retropubic mid-urethral sling (TVT) placement, attributable to a prior inguinal hernia repair mesh. This finding underscores the importance of a detailed surgical history, with particular emphasis on prior pelvic or groin procedures. In such cases, preoperative imaging should be considered to anticipate potential technical interference.
